# Supplementary material for: Mucopolysaccharidosis-Plus Syndrome: Report on a Polish Patient with a Novel VPS33A Variant with Comparison with Other Described Patients
Source: Int J Mol Sci. 2022 Sep 28;23(19):11424. doi: 10.3390/ijms231911424 (PMC9570340; doi:10.3390/ijms231911424)
Supplement: Supplementary file 1 [file ijms-23-11424-s001.zip › ijms-1903347-supplementary.pdf]

Supplementary Material

MS instrument parameters for panel 1.

Curtain Gas (CUR)	30 L/h
Collision Gas (CAD)	Medium
Ion Spray Voltage	5500 V
Temperature (TEM)	480^{0}C
Ion Sourse Gas 1 (GS1)	50 L/h
Ion Sourse Gas 1 (GS2)	65 L/h
Dwell time	18 ms
Declustering potential (DP)	40 V
Entrance Potential (EP)	10 V
Collision Cell Exit potential (CXP)	10 V

MRM parameters for panel 1.

	Precursor ion (*m/z*)	Product ion (*m/z*)	CE (V)
GALC-P	412.20	264.20	30
GALC-IS	417.20	264.20	30
GAA-P	498.20	398.20	32
GAA-IS	503.20	403.20	32
GLA-P	484.20	384.20	32
GLA-IS	489.20	389.20	32
ABG-P	384.20	264.20	28
ABG-IS	391.20	271.20	28
ASM-P	398.20	264.20	30
ASM-IS	405.20	264.20	30
IDUA-P	426.20	317.20	25
IDUA-IS	431.20	322.20	25

P, product of enzyme reaction
IS, internal standard.
DP, declastering potencial
CE, collision energy

HPLC method for panel 1.

Column	XBridge BEH C18 (Waters, USA).<br>Particle diameter: 3.5 µm<br>Internal diameter: 2.1 mm<br>Length: 50 mm
Column temperature	60°C
Wash solvent	80% Methanol in water
Mobile phase A	0.2% formic acid in water
Mobile phase B	0.2% formic acid in acetonitrile
Gradient (% B)	0 - 0.50 min: 10% B – 98% B<br>0.50 - 2.00 min: 98% B<br>2.00 – 3.00 min: 10% B
Flow rate	0.4 mL/min
Injection volume	10 µL
Autosampler temperature	15°C

MS instrument parameters for panel 2.

Curtain Gas (CUR)	20 L/h
Collision Gas (CAD)	Medium
Ion Spray Voltage	5500 V
Temperature (TEM)	600^{0}C
Ion Sourse Gas 1 (GS1)	50 L/h
Ion Sourse Gas 1 (GS2)	65 L/h
Dwell time	20 ms
Entrance Potential (EP)	10 V

Collision Cell Exit potential (CXP)	10 V

MRM parameters for panel 2.

	Precursor ion (*m/z*)	Product ion (*m/z*)	DP (V)	CE (V)
NAGLU-P	420.30	311.30	65	21
NAGLU-IS	423.30	314.30	65	21
GALNS-P	685.40	373.30	80	35
GALNS-IS	690.40	378.30	80	35
ARSB-P	657.40	345.20	80	33
ARSB-IS	662.40	350.30	80	33
GLB1-P	436.30	336.30	50	21
GLB1-IS	439.30	339.30	50	21
GUSB-P	434.30	325.30	80	34
GUSB-IS	439.30	330.30	80	34
I2S-P	644.40	359.30	80	30
I2S-IS	649.30	364.30	80	30
TPP1-P	350.30	250.30	36	20
TPP1-IS	359.30	251.30	36	20

P, product of enzyme reaction
IS, internal standard.
DP, declastering potencial
CE, collision energy

HPLC method for panel 2.

Column	Fusion-RP (Phenomenex, USA).
	Particle diameter: 4.0 µm
	Internal diameter: 2.1 mm
	Length: 50 mm
Column temperature	60°C
Wash solvent	80% Methanol in water
Mobile phase A	0.2% formic acid in water
Mobile phase B	0.2% formic acid in acetonitrile
Gradient (% B)	0 – 2.00 min: 10% B – 80% B
	2.00 - 2.50 min: 80% B
	2.50 – 3.50 min: 10% B
Flow rate	0.5 mL/min
Injection volume	5 µL
Autosampler temperature	15°C
